# Basic Medium Heterogeneous Solution Synthesis of α-MnO_2_ Nanoflakes as an Anode or Cathode in Half Cell Configuration (vs. Lithium) of Li-Ion Batteries

**DOI:** 10.3390/nano8080608

**Published:** 2018-08-09

**Authors:** Kyungho Kim, Geoffrey Daniel, Vadim G. Kessler, Gulaim A. Seisenbaeva, Vilas G. Pol

**Affiliations:** 1 Materials Science and Engineering, Purdue University, West Lafayette, IN 47907, USA; kim1797@purdue.edu; 2 Department of Biomaterials and Technology, Swedish University of Agricultural Sciences, Box 7008, SE-75007 Uppsala, Sweden; geoffrey.daniel@slu.se; 3 Department of Molecular Sciences, Swedish University of Agricultural Sciences, Box 7015, SE-75007 Uppsala, Sweden; vadim.kessler@slu.se; 4 Davidson School of Chemical Engineering, Purdue University, West Lafayette, IN 47907, USA

**Keywords:** α-MnO_2_, nanostructured matrix, base medium fabrication, electrode materials, alkali metal ion battery

## Abstract

Nano α-MnO_2_ is usually synthesized under hydrothermal conditions in acidic medium, which results in materials easily undergoing thermal reduction and offers single crystals often over 100 nm in size. In this study, α-MnO_2_ built up of inter-grown ultra-small nanoflakes with 10 nm thickness was produced in a rapid two-step procedure starting via partial reduction in solution in basic medium subsequently followed by co-proportionation in thermal treatment. This approach offers phase-pure α-MnO_2_ doped with potassium (cryptomelane type K_0.25_Mn_8_O_16_ structure) demonstrating considerable chemical and thermal stability. The reaction pathways leading to this new morphology and structure have been discussed. The MnO_2_ electrodes produced from obtained nanostructures were tested as electrodes of lithium ion batteries delivering initial discharge capacities of 968 mAh g^−1^ for anode (0 to 2.0 V) and 317 mAh g^−1^ for cathode (1.5 to 3.5 V) at 20 mA g^−1^ current density. At constant current of 100 mA g^−1^, stable cycling of anode achieving 660 mAh g^−1^ and 145 mAh g^−1^ for cathode after 200 cycles is recorded. Post diagnostic analysis of cycled electrodes confirmed the electrode materials stability and structural properties.

## 1. Introduction

Lithium ion batteries (LIB) have attracted huge attention in the battery industry since their introduction in the early 1990s by Sony. The growing attention to large intermittent devices and electric vehicles requires high energy and high power density batteries. Thereby, developing LIB with good performance and excellent stability becomes essential. Metal oxides are studied as electrode materials for LIB due to their high theoretical capacity compared to graphite [1,2,3,4]. Among various transition metal oxides, manganese-based oxides (MnO, Mn_2_O_3_, Mn_3_O_4_, and MnO_2_) have attracted huge interest as electrode materials for their high theoretical capacity, eco-friendliness, cost-effectiveness, and natural abundance [5]. Based on the conversion reaction of each manganese oxide, the theoretical capacity is 750, 936, 1018, and 1230 mAh g^−1^ for MnO, Mn_3_O_4_, Mn_2_O_3_, and MnO_2_, respectively. Traditionally, MnO_2_ is applied as a material for primary lithium batteries and capacitors [6,7]. Recently, the interest in lithium-ion batteries has increased as well for their high theoretical capacity among four different types of manganese oxides [8,9].

However, MnO_2_ as an electrode material in secondary ion batteries has been hindered by the large volume expansion, poor conductivity, and poor reversibility [5,8]. To address such an issue, several strategies have been suggested including MnO_2_ composite material and fabricating nano-structured MnO_2_. Li et al. wrapped MnO_2_ with graphene to resolve the conductivity issue [10]. The initial capacity was 1200 mAh g^−1^ and a stable capacity of 600 mAh g^−1^ at 400 mA g^−1^ current density was maintained. MnO_2_ composite with improved conductivity has been realized by using graphene [11], carbon nanotube [12], and other metal oxides [13,14,15]. The size, crystallinity, and morphology control of MnO_2_ are important factors that influence the performance. Thereby, it is essential to choose the proper synthesis method. Numerous reports use the acidic medium fabrication method to prepare MnO_2_. Chen et al. have prepared MnO_2_ nano-rods using KMnO_4_ and HCl via the hydrothermal method [8]. The prepared nano-rods have shown an initial capacity of 1200 mAh g^−1^. However, the cycle performance was unstable. Another example of using acidic medium in hydrothermal process was reported by Feng and coworkers [16]. Despite the high initial capacity (1400 mAh g^−1^), the capacity drops to 600 mAh g^−1^ at second cycle. Li and coworkers prepared hollow urchin-like MnO_2_ via mixture of KMnO_4_ and sulfuric acid [17]. The hollow urchin morphology is obtained by the Ostwald ripening and delivered an initial capacity of 746 mAh g^−1^. Still, the cycle retention was 64% after 40 cycles. In addition to anode, there have also been few works on MnO_2_ as cathode material reported. Abuzeid et al. prepared MnO_2_ by using KMnO_4_ and extracts of green tea [5]. The prepared material showed an initial capacity of 236 mAh g^−1^ and 73% cycle retention after 20 cycles.

The majority of research used acidic medium for fabricating MnO_2_. Especially, hydrothermal approaches in acidic medium for preparation of the MnO_2_ are well reported [18,19]. In realization of this approach, a broad variety of precursors and conditions have been evaluated. In particular, acidic leaching of Mn(II) with H_2_SO_4_ from Mn_2_O_3_ has been reported [20,21]. Even decomposition of KMnO_4_ solutions in acidic medium at controlled low pH has been investigated [22]. The most thoroughly investigated tracks have been, however, the reduction of KMnO_4_ by HCl or by MnSO_4_ in acidic medium [23,24]. In addition, the use of organic polymers for permanganate reduction has been investigated [25,26]. Other approaches involved oxidation of MnSO_4_ solutions by potassium persulfate, K_2_S_2_O_8_, or by ozone [16]. Oxidation by atmospheric oxygen has also been used in the co-precipitation approach in basic medium that was using MnSO_4_ and MnC_2_O_4_ as Mn-sources. Despite acidic medium based MnO_2_ showed promising results, acidic medium results in partial reduction of Mn(IV) in the produced MnO_2_ nanostructures, leading potentially to lower capacity and instability in cycling of the produced electrode materials.

In this work, we report a successful fabrication of α-MnO_2_ nano flakes by reduction approaches in basic medium, securing preservation of the oxidation state +4. An unusual approach based on seeding of α-MnO_2_ through thermal treatment-induced interaction of amorphous MnO_2_, precipitated from permanganate by ammonia, with fine powder of stoichiometric amounts of Mn(II) carbonate resulted in interpenetrated network of phase-pure nanocrytalline material. Table 1 shows the comparison of current work to other’s work on MnO_2_ as electrode material. The structure analysis showed successful fabrication of nano-MnO_2_ particles. In addition, the preservation of MnO_2_ has been observed by Fourier Transformed Infrared Spectroscopy (FTIR) and Thermogravimetric Analysis (TGA). Basic medium based α-MnO_2_ showed successful results as electrode material for LIB and revealed a stable capacity of 690 mAh g^−1^ and 150 mAh g^−1^ at 100 mA g^−1^, respectively. The behavior of α-MnO_2_ was discussed by the voltage profile and cyclic voltammetry curves. To confirm that basic medium fabrication is compatible to conventional acidic medium based MnO_2_, the capacity values are compared.

## 2. Experimental

### 2.1. Synthesis of MnO_2_

All chemicals purchased from Sigma-Aldrich (Copenhahen, Denmark) were used without further purification. The synthesis was conducted under an ambient atmosphere. For preparation of α-MnO_2_ nano flakes, 0.2 g KMnO_4_ (1.28 mmol) was dissolved in 10 mL deionized water and a slurry of 0.5068 g of MnCO_3_·H_2_O (3.84 mmol) in 15 mL 25% NH_3_ solution was added. Constant stirring for 1 h at 80 °C were applied and then additional 5 mL 25% NH_4_OH were added and continued stirring for 1 h. The resulting brown precipitate was collected by decantation, washed and centrifuged with three portions of 10 mL of distilled water and dried in air overnight followed with heat treatment at 400 °C in air (heating up with the rate of 10 °C/min to 400 °C from room temperature and keeping then at this temperature for 4 h. The time was optimized experimentally by ex-situ X-ray Diffraction (XRD, Stockholm, Sweden) studies after different annealing times—2, 3, 4 and 6 h). The yield of resulting greyish black powder is 0.3781 g (86%).

### 2.2. Material Characterization

XRD studies of the powders were carried out with D8 SMART CCD Apex-II diffractometer (Bruker Nordic, Stockholm, Sweden) in rotation mode. SEM energy-dispersive X-ray spectroscopy (EDS) investigation was performed using Hitachi TM-1000-µ-DEX instrument (Spectral Solutions AB, Stockholm, Sweden). TEM investigation was made with Phillips CM12 instrument (Philips, Sweden). AFM studies have been performed with Bruker FastScan with ScanAsyst microscope (Blue Scientific, Stockholm, Sweden). FTIR studies were made with Spectrum 100 instrument (Perkin-Elmer, Upplands Väsby, Sweden). Thermogravimetric investigation was carried out with Pyris-1 thermobalance (Perkin-Elmer, Upplands Väsby, Sweden).

### 2.3. Electrochemistry

The electrochemical properties of α-MnO_2_ electrode materials were tested in half cells configuration with lithium-metal as counter-electrode as an anode (loading mass: 0.3 mg cm^−2^) and cathode (loading mass: 0.3 mg cm^−2^). 70% of prepared α-MnO_2_ was mixed with 20% conducting carbon (Timcal Super P, MTI Corporation, Richmond, CA, USA) and 10% PVDF (MTI Corporation, Richmond, CA, USA) binder in *N*-methyl-2-pyrrolidone (NMP) solvent. Obtained slurry was coated on aluminum (for cathode) and copper (for anode) current collectors. After drying overnight in a vacuum oven set at 80 °C, the prepared working electrode is assembled into 2032 coin cells (CR 2032) inside a glove box. Lithium foil (MTI Corporation, Richmond, CA, USA) was used as a counter-electrode, Cellgard 2500 (Charlotte, NC, USA) as a separator, and 1 M lithium hexafluorophosphate (LiPF_6_) salt in ethylene carbonate (EC)/diethyl carbonate (DEC) (Sigma Aldrich, St. Louis, MO, USA, 1:1 volume ratio) as an electrolyte. Galvanostatic charge-discharge profile was measured in the potential range of 0.01 V to 2 V for anode and 1.5 V to 3.5 V for cathode (BST8-MA, MTI, Richmond, CA, USA). In addition, cyclic voltammetry was performed by Gamry Reference 600+ equipment (Warminster, PA, USA) at a scanning rate of 0.1 mV s^−1^.

## 3. Results and Discussion

### 3.1. Synthesis and Structure of the Material

In preparation of manganese dioxide in basic medium, the reduction of potassium permanganate with concentrated ammonia was conducted. The proposed route involves setting together a solid phase, MnCO_3_(s), together with the solution of KMnO_4_ and NH_3_(aq). The reaction in the liquid phase provides amorphous K*_x_*MnO_2+*y*_ (and N_2_(g) nitrogen), with oxidation state for Mn higher than +IV. In contact with MnCO_3_, this results apparently in seeding of MnO_2_(s) on the surface of the carbonate crystals. In subsequent thermal treatment, the whole mass is transformed into small-size α-MnO_2_ crystallites, both the amorphous part crystallizes with release of adsorbed water and breakdown of surface hydroxide groups and the solid MnCO_3_ is broken down with release of CO_2_. It is noteworthy that the thermal treatment of MnCO_3_ only gives as revealed by XRD in this work a complex mixture of Mn_2_O_3_, Mn_3_O_4_ and MnO_2_ (see Supplementary Figure S1) and also the heat-treatment of the amorphous mass, resulting from reduction of KMnO_4_ by NH_3_ on reflux in concentrated solution offers a complex mixture of phases after heat-treatment at over 600 °C (Figure S2). It is the combination of the two systems that results in practically phase-pure MnO_2_. Addition of a stoichiometric amount of manganese (II) carbonate, MnCO_3_, provides the necessary reducing agent not causing inclusion of other transition metal cations [27].

**Figure 1:**
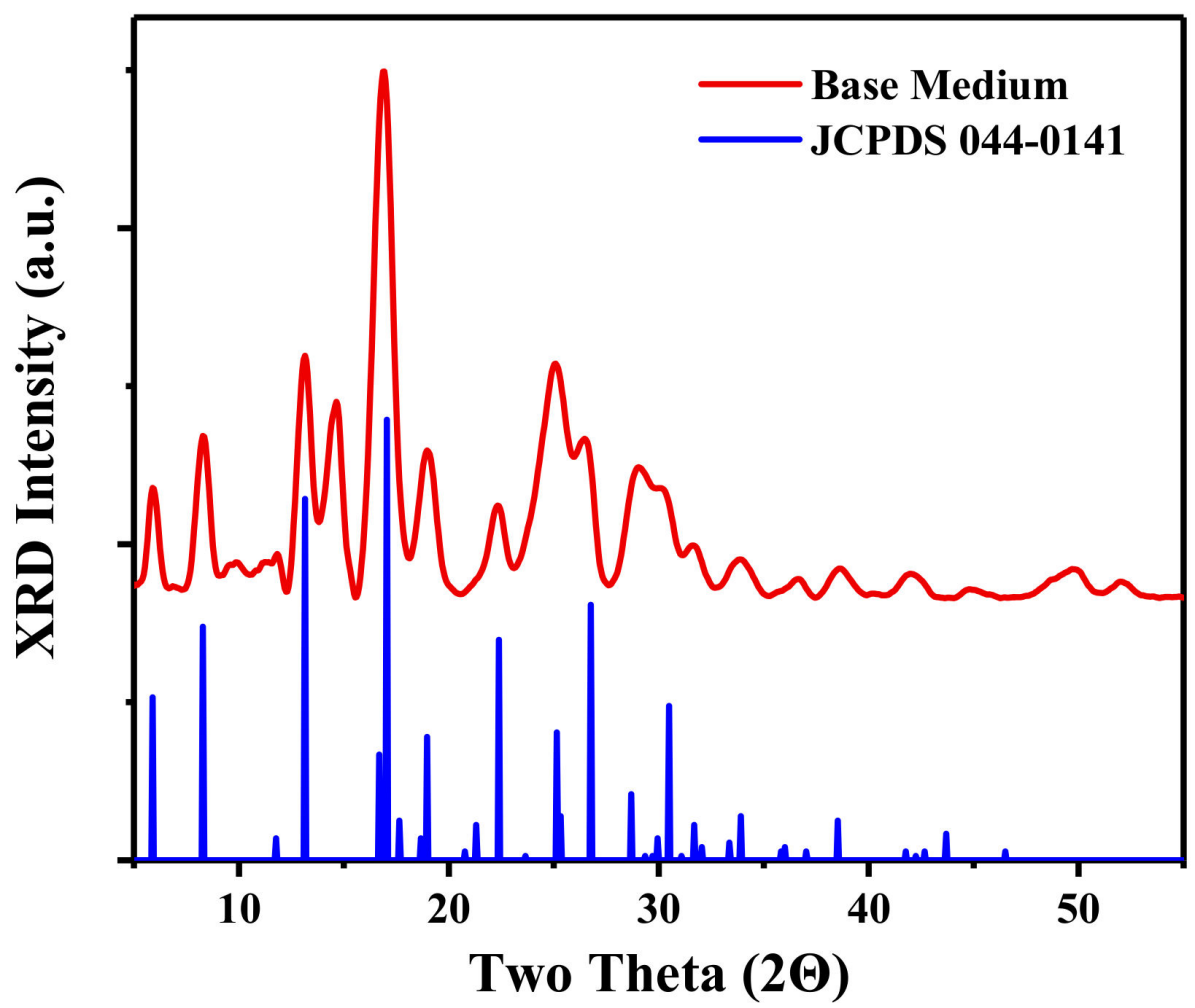
XRD pattern for the produced α-MnO_2_ nanomaterial.

**Figure 2:**
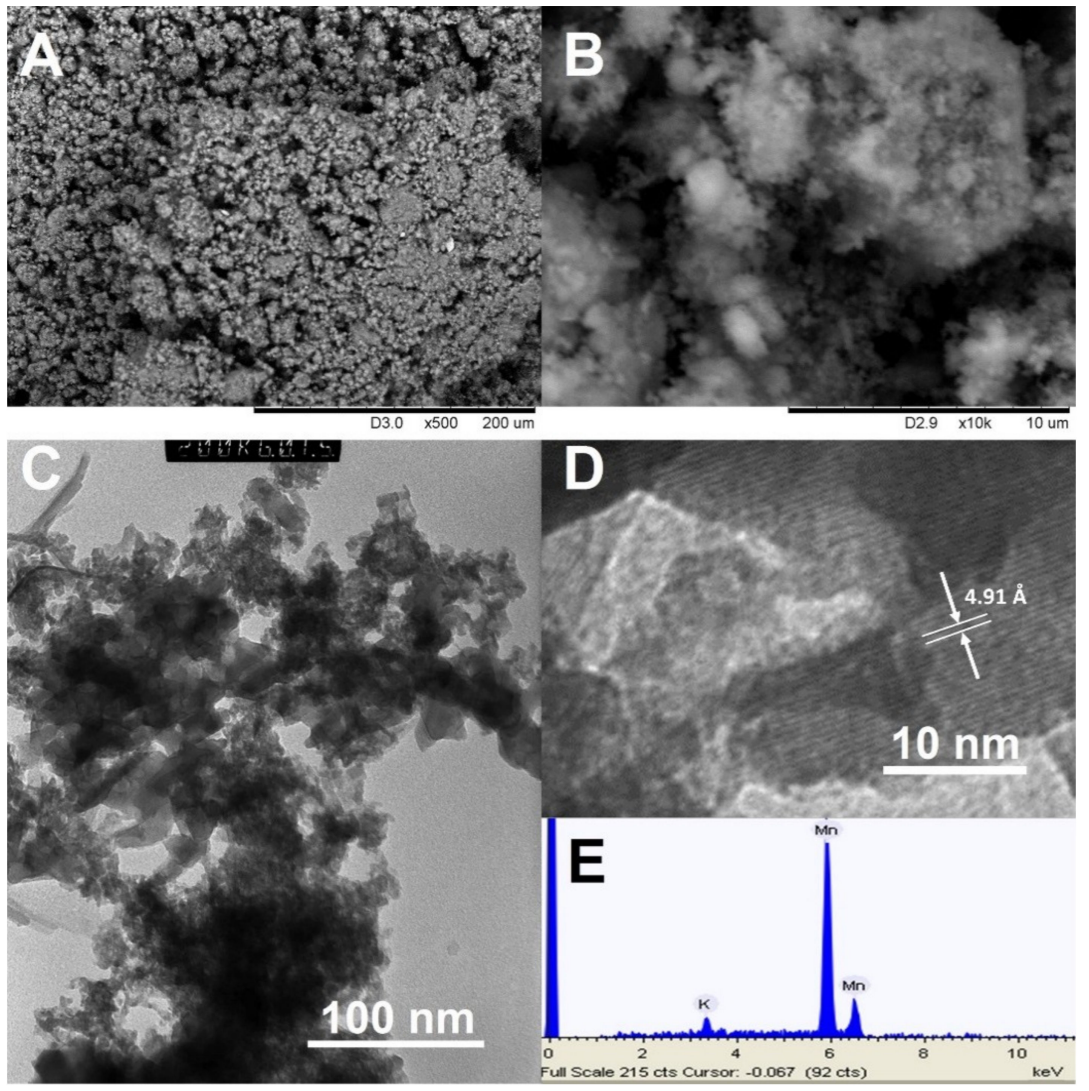
SEM (**A**,**B**) and TEM (**C**,**D**) images and EDS analysis of the α-MnO_2_ obtained (**E**).

The product obtained from the initial reaction step in solution revealed a low-intensity X-ray diffraction pattern corresponding to MnCO_3_ together with an amorphous phase (see Supplementary Figure S3). Subsequent thermal treatment in 2 h at 400 °C led to a practically amorphous material according to XRD, while further treatment in 2 h resulted in a crystalline sample with XRD pattern corresponding to α-MnO_2_ phase (Figure 1). The average crystallite size calculated from Scherrer equation using the most intense line at 2θ = 16.987° (with Miller indices hkl = 211) is 108 nm. This is as stated only an average value as both smaller and bigger crystallites (over 100 nm) could be observed by TEM (see Figure 2). The peak at 2θ = 14.707° is corresponding to minor impurity of the residual MnCO_3_ (the most intense hkl = 104 line).

The SEM images (see Figure 2A,B) show strongly aggregated small particles forming low mass density solid material. The morphology of the prepared MnO_2_ revealed by TEM shows a flake-like structure with 0.491 nm 200 plane fringe pattern, which agrees to the Cryptomelane type α-MnO_2_ structure (Figure 2D). Low-charge cations are required to stabilize the Cryptomelane MnO_2_ structure—the formula of the natural Cryptomelane mineral is KMn^IV^_7_Mn^III^O_16_ [28]. The α-MnO_2_ obtained in this work is apparently stabilized by the minor residual content of potassium in its composition. The Mn to K ratio was determined by EDS analysis and was found to be Mn:K = 32:1, i.e., only 25% of the additional positions in the Cryptomelane structure are thus occupied by the K-cations in the classic mineral structure, which here can be formulated as K_0.25_Mn_8_O_16_.

The flakes are connected in a very open interpenetrated network (Figure 2C) that is apparently formed through the thermal decomposition process associated with evolution of gases—both CO_2_ from MnCO_3_ crystals and water vapor from K-containing amorphous hydrated precipitate surrounding the carbonate crystals in the initial product of solution reaction. The process is straightforward and easy to carry out. The Brunauer-Emmett-Teller (BET) analysis was conducted to further confirm the interconnected structure (Figure S4). The pore distribution profile shows existence of various pore size in the prepared materials. This is attributed to the pore existing between interconnected MnO_2_ nanoparticles. In addition, the surface area (61.5 m^2^ g^−1^) is relatively higher than reported bulk MnO_2_ nanoparticles, which supports the interconnected structure as well [5,21].

**Scheme 1:**
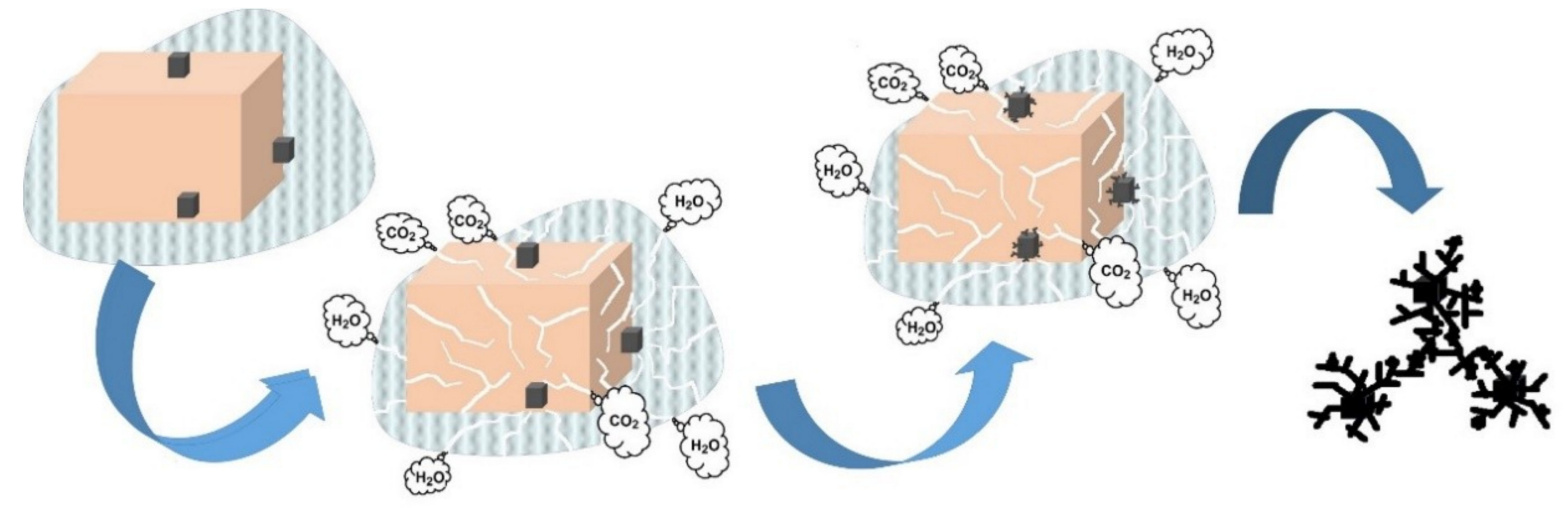
Reaction pathway in producing hollandite type α-MnO_2_ phase.

**Figure 3:**
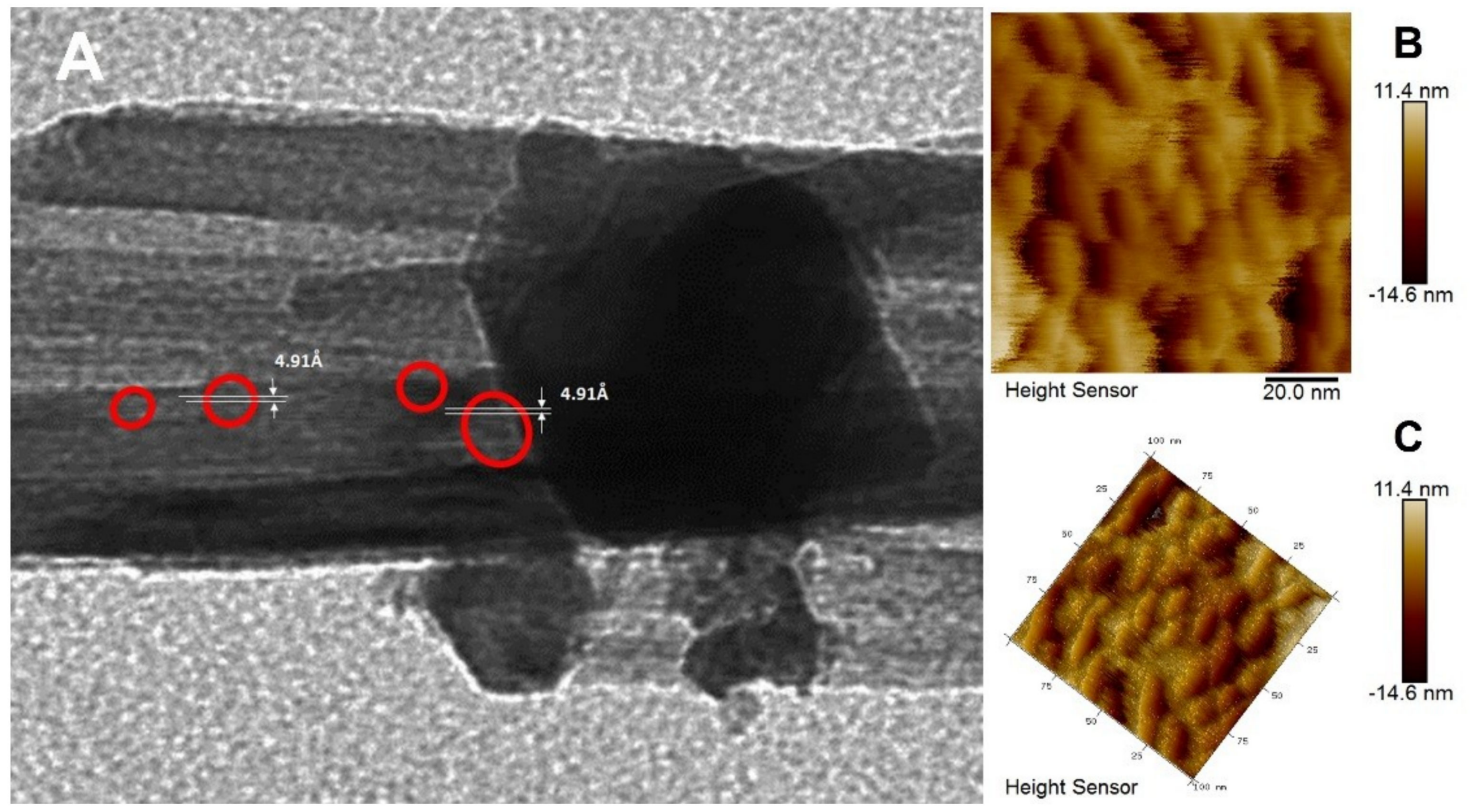
TEM (**A**) and AFM (**B**,**C**) images of the material obtained in the course of thermal transformation of the reaction mixture into α-MnO_2_.

Direct confirmation to the proposed mechanism could be obtained in the high resolution TEM and AFM studies of the intermediate reaction mixtures. As can be seen in Figure 3, the amorphous flakes containing K-cations and produced by decomposition of KMnO_4_ in the presence of ammonia commence to crystallize forming nuclei of the Cryptomelane type phase. The crystallites of MnCO_3_ are turned amorphous by the thermal treatment but keep their shape. The AFM images (Figure 3B,C) show clearly the rhombic shape blocks derived from the initial MnCO_3_ crystallites embedded into a less dense matrix. The final material is produced by crystallization accompanied with diffusion of potassium into these bigger blocks as the final material is single phase and has uniform potassium distribution in it according to EDS. The overall mechanism of the process can thus be described by Scheme 1.

The occurring chemical transformations can be described by the following reaction equations:

3KMnO_4_(aq) + (4 − 2*x*)NH_3_(aq) → 3KMnO_2+*x*_(s) + (2 − *x*)N_2_(g) + 6H_2_O (1)

MnCO_3_(s) → MnO_2−*y*_ + CO_2_(g) (2)

KMnO_2+*x*_(s) + 3MnO_2−*y*_(s) → K_0.25_MnO_2_(s) (3)

### 3.2. Stability Test of Produced α-MnO_2_

FTIR (Figure S5) shows the chemical information of samples kept at ambient condition. Minor degree of hydration (δ(O–H) at about 1600 and ν(O–H) at 3700 cm^−1^) and the network of metal-oxygen bonds present as characteristic bands (around 536 and 585 cm^−1^).

The TGA (Figure S6) shows the loss of hydrating water starting at room temperature and finishing at about 200 °C with the loss of 2.4–2.7% of the total weight in both air and nitrogen atmosphere. This process is followed by thermal decomposition with reduction of MnO_2_ into Mn_3_O_4_ in air at 500–620 °C (corresponding weight loss 6.1%) and into first Mn_3_O_4_ at 470–560 °C (weight loss of about 6%) and then Mn_2_O_3_ at 660–800 °C (further weight loss of about 3%) in N_2_ atmosphere. This confirms that MnO_2_ was produced when the heating was carried out to 400 °C with subsequent annealing and shows considerable thermal stability of the obtained material.

### 3.3. Electrochemical Performance

**Figure 4:**
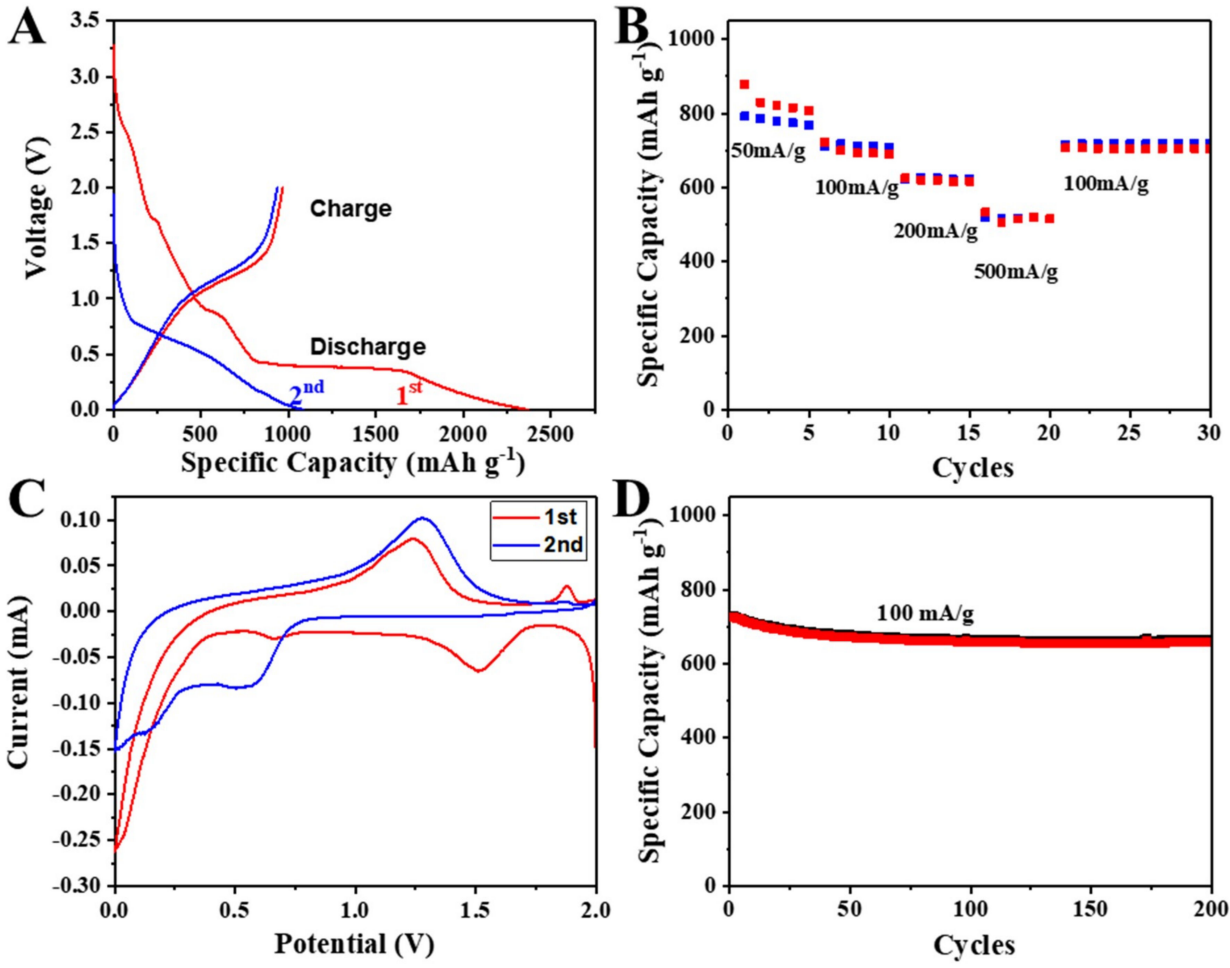
Electrochemical data of α-MnO_2_ anode electrode. (**A**) charge-discharge profiles for first two cycles, (**B**) rate study, (**C**) cyclic voltammetry curve, and (**D**) constant current (100 mA g^−1^) cycling.

The α-MnO_2_ electrode was assembled and tested as anode and cathode separately vs. lithium (half cells) for lithium ion batteries. Figure 4A shows the voltage profiles of MnO_2_ for the initial two cycles at a current density of 20 mA g^−1^. It exhibits an initial discharge and charge capacity as 2369 and 968 mAh g^−1^, respectively, with a 59.1% first cycle Coulombic efficiency. The loss of capacity at the first cycle is attributed to the irreversible conversion reactions of lithium and MnO_2_ and the formation of solid electrolyte interphase (SEI) layer, which is obvious by the first cycle voltage profile slope and plateau in 1.5 to 0.7 V range [8]. The cyclic voltammetry (CV) was conducted to understand the kinetics (Figure 4C). The initial two cycles are scanned at a range of 0.01 to 2.0 V at a 0.1 mV s^−1^ scan rate. The CV curve for the first cycle shows a cathodic peak near 1.5 V which is not observed at the second cycle. The SEI layer and the irreversible conversion reaction between lithium and MnO_2_ shows the observed cathodic peak [16]. In addition, a broad peak appearance from 1.5–0.5 V corresponds to the reduction of MnO_2_ to Mn^2+^, and the peak near 0.1 V corresponds to the reduction of Mn^2+^ to metallic Mn^0^, which is easily found in other transition metal compounds for lithium ion batteries. An anodic peak appearance at 1.30 and 2.0 V is a response to the oxidation of Mn^0^ and Mn^2+^, respectively [8]. A shift in the second cycle explains a possible structure change induced by the formation of metallic manganese.

Figure 4B explains the rate performance of MnO_2_ as an anode at different current density. The rate study was conducted at various current densities, from 50 mA g^−1^ to 500 mA g^−1^. Stable capacities of 791 mAh g^−1^ and 517 mAh g^−1^ at current densities 50 mA g^−1^ and 500 mA g^−1^, respectively is obtained. The comparison of initial and final capacities at current densities 100 mA g^−1^ (711 mAh g^−1^ vs. 716 mAh g^−1^) shows excellent rate recovery after high rate performance. The cycle retention is 91% over 200 cycles (Figure 4D, 0.045% per cycle) which is relatively stable compared to other reports on MnO_2_ as anode (Table 1).

**Figure 5:**
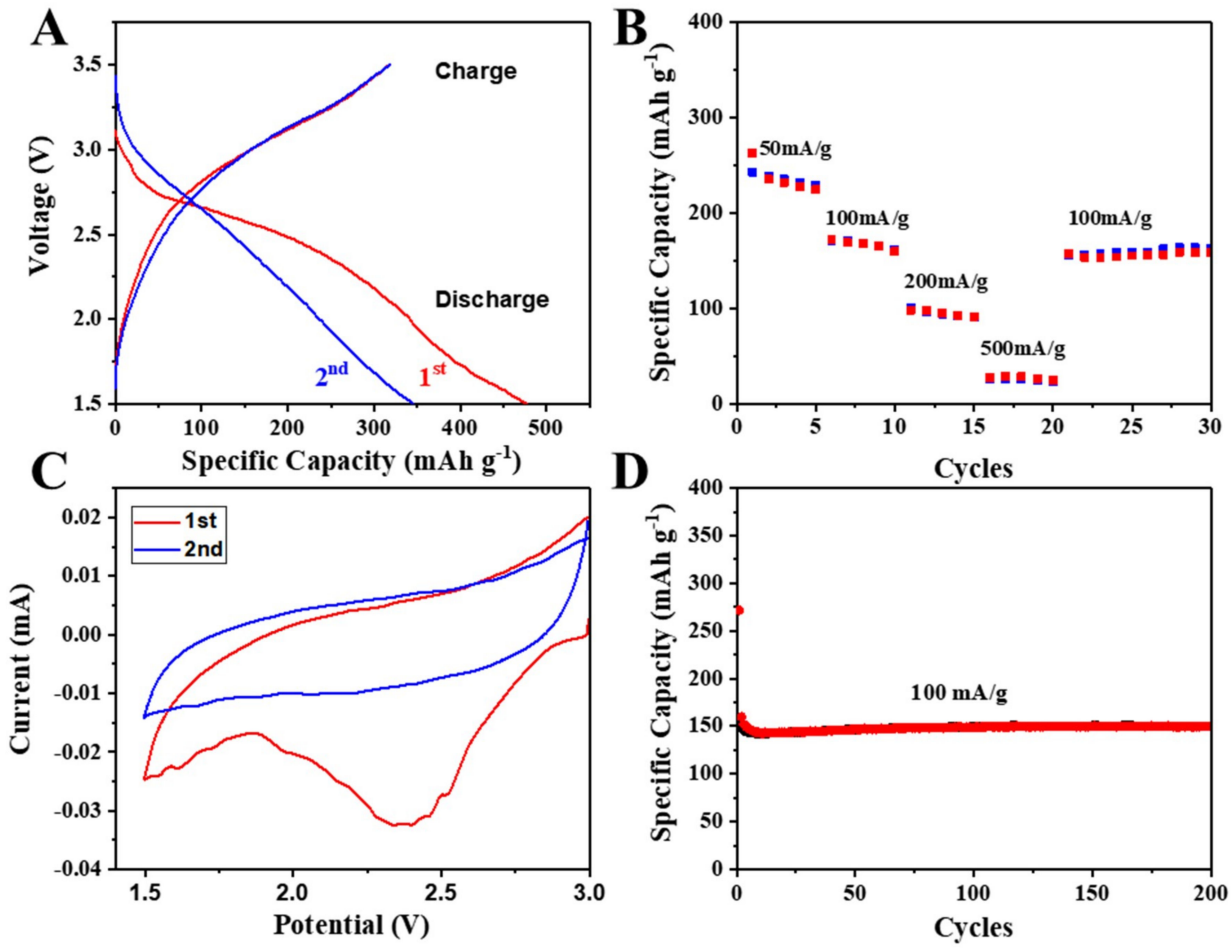
Electrochemical data of α-MnO_2_ cathode electrode. (**A**) charge-discharge profile, (**B**) rate study, (**C**) cyclic voltammetry curve, and (**D**) long cycle study at current density of 100 mA g^−1^.

Numerous reports also focused on the possibility of using acid medium fabricated α-MnO_2_ as cathodic materials. To further ascertain that basic medium fabrication produced α-MnO_2_ is successful, its cathodic activities are tested at higher potential and compared (Table 1). The charge-discharge profile at a current density of 20 mA g^−1^ displayed an initial discharge and charge capacity as 477 and 317 mAh g^−1^, respectively, with a 33.4% capacity drop (Figure 5A). A plateau region occurring at a broad range over 2.5 to 2.2 V is attributed to the reduction of Mn^4+^ ions. The cyclic voltammetry (CV) shows analogous result to voltage profile, exhibiting an anodic peak at a similar range (oxidation of Mn^2+^ to Mn^4+^) with voltage profile (Figure 5C). The discharge capacity of 171 mAh g^−1^ recovered to 155 mAh g^−1^ after rate study (91% recover for cathode). The low capacity number at high current density can be explained by the sluggish kinetics of Li ion. Numerous reports compared the influence of Li ion conductivity on rate performance, showing low conductivity results in low rate performance [29,30]. The cycle retention of cathode is measured and displayed in Figure 5D. The capacity retention is 93% over 200 cycles (0.035% decrease per cycle).

**Figure 6:**
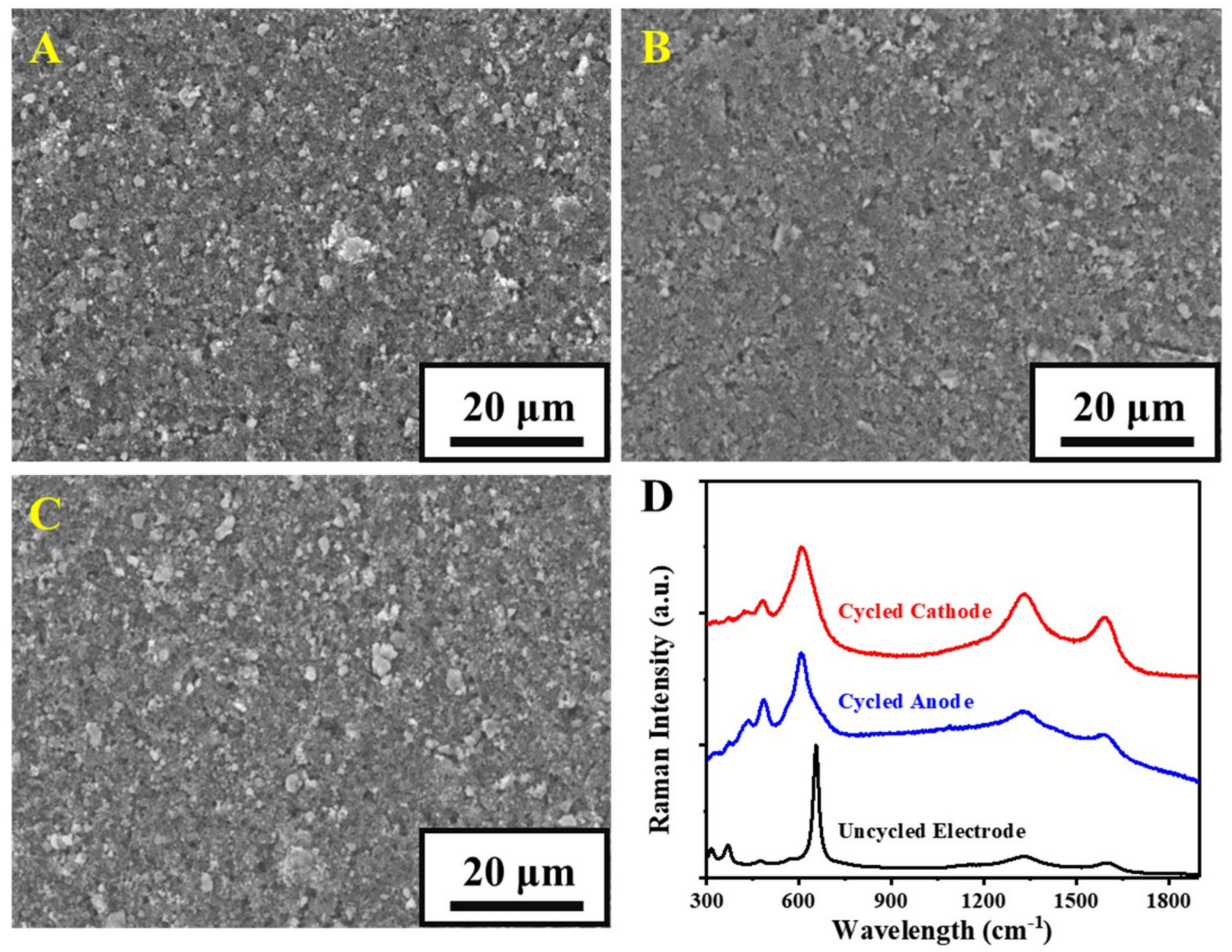
SEM images of (**A**) un-cycled electrode, (**B**) anode cycled for 200 cycles, (**C**) cathode cycled for 200 cycles, and (**D**) Raman spectra of each electrode.

Post-diagnostic analysis was carried out to determine whether the basic medium synthesized α-MnO_2_ is stable after long-term cycling (200 cycles). SEM and Raman analysis were performed on before and after cycled electrodes (both anode and cathode). The as-prepared electrode is composed of α-MnO_2_ (active material, conductive carbon (Super P), and PVDF binder (Figure 6A). It is observed that a uniform, homogeneous electrode is fabricated using α-MnO_2_ powder. The SEM image of cycled anode (Figure 6B) and cathode electrode (Figure 6C) shows that there is no obvious physical change in laminate. Raman spectroscopy of pristine electrode shows a peak positioned at 650, 1330, and 1598 cm^−1^, indicating presence of α-MnO_2_ (650 cm^−1^) and super P (conductive carbon), respectively [31,32,33]. For both cycled electrodes, the new Raman band appeared and the peak shows a broad feature compared to pristine electrode Raman peak (Figure 6D). The broad peak can be explained by the partial disordered phase of MnO_2_ after lithiation and delithiation process. After cycling, new Raman band appears at 484 and 430 cm^−1^, which agrees to reported band position of Li-birnessite in Raman [34]. The similarity of Raman spectra suggests that during the repeated lithiation and delithiation, the hollandite MnO_2_ structure partially transformed to layered birnessite-type Li-MnO_2_ [35,36].

## 4. Conclusions

The newly proposed approach, combining solution redox reaction and seeding on the surface of introduced MnCO_3_ solid, offers a facile approach to α-MnO_2_ phase stabilized by residual potassium content and shaped as nano flakes. Compared to acidic medium synthesis, this study shows advantages in, on one hand, operating with a phase-pure Mn(IV) derived material, and, on the other hand, exploiting a nanostructure of small particles with an average size of 10 nm, connected into an interpenetrated network created by the thermal decomposition synthesis associated with evolution of gases. A stable capacity of 791 and 243 mAh g^−1^ at 50 mA g^−1^ were obtained for α-MnO_2_ tested as anode and cathode, respectively. The rate performance was stable as well, showing 718 and 155 mAh g^−1^ at 100 mA g^−1^ for anode and cathode, respectively. From this study, we demonstrated that MnO_2_ obtained from basic medium is a promising electrode material for anode as well as cathode. This method can be further utilized in preparing materials that are sensitive to acid medium.

## Figures and Tables

**Table 1:** Comparison of current work to literature reports on MnO_2_ in lithium ion batteries.

References	Synthesis Method	Initial Capacity (Current Density)	Cycle Retention	RateCapacity	Electrode
Chen [8]	KMnO_4_ + HCl Hydrothermal	1119 mAh g^−1^ (100 mA g^−1^)	96% (100 cycles)	1000 mAh g^−1^ (200 mA g^−1^)	Anode
Vernardou [1]	KMnO_4_ + Nitric Acid Hydrothermal	943 mAh g^−1^	N/A	N/A	Anode
Walanda [20]	Hydrothermal of Mn_2_O_3_ in H_2_SO_4_	~1100 mAh g^−1^ (20 mA g^−1^)	N/A	N/A	Anode
Li [17]	MnSO_4_ + H_2_SO_4_ Hydrothermal	750 mAh g^−1^ (270 mA g^−1^)	64% (40 cycles)	N/A	Anode
Dai [18]	MnSO_4_ + H_2_SO_4_ Ozone Oxidation	200 mAh g^−1^ (20 mA g^−1^)	82.5% (20 cycles)	N/A	Cathode
Abuzeid [5]	KMnO_4_ + H_2_SO_4_ green-like synthesis method	236 mAh g^−1^ (20 mA g^−1^)	73% (20 cycles)	75 mAh g^−1^ (200 mA g^−1^)	Cathode
Our Study	KMnO_4_ + NH_4_OH Stirring and precipitation	800 mAh g^−1^ (50 mA g^−1^)	91% (200 cycles)	600 mAh g^−1^ (200 mA g^−1^)	Anode
		250 mAh g^−1^ (50 mA g^−1^)	93% (200 cycles)	100 mAh g^−1^ (200 mA g^−1^)	Cathode

## Supplementary Materials

The following are available online at http://www.mdpi.com/2079-4991/8/8/608/s1, Figure S1: XRD pattern of decomposition products of MnCO_3_ with characteristic peaks for Ramsdellite MnO_2_ (ICPDS Card 82-2169), Mn_5_O_8_ (39-1218), Hausmannite Mn_3_O_4_ (80-382), Akhtenskite-MnO_2_ (30-0820) and Rhodochrosite MnCO_3_ (86-0173) marked in the image, Figure S2: XRD of the material obtained by heat-treatment of the precipitate from the reaction KMnO_4_ with NH3 in aqueous medium at 600 °C in 2 h in air with characteristic peaks indicated for K_0.5_Mn_2_O_4.1.5_H_2_O (JCPDS Card 42-1317), Crypromelane KMn_8_O_16_ (34-0168) and K_6_Mn_2_O_6_ (ICDD PDF 01-070-1271), Figure S3: XRD of the initial product of solution synthesis featuring the pure MnCO_3_ phase ICPDS Card No. 86-0173, Figure S4: BET analysis of (a) N2 absorption isotherm and (b) pore size distribution profile of prepared α-MnO_2_ nanomaterial, Figure S5: FTIR analysis for produced α-MnO_2_ nanomaterial, Figure S6: TGA (solid line) and DTG (dashed line) analysis of produced α-MnO_2_ nanomaterial in the air (A) and in the nitrogen (B) atmosphere.

## References

[1] Thirugunanam L., Kaveri S., Etacheri V., Ramaprabhu S., Dutta M., Pol V.G. (2017). Electrospun nanoporous TiO_2_ nanofibers wrapped with reduced graphene oxide for enhanced and rapid lithium-ion storage. Mater. Charact..

[2] Henzie J., Etacheri V., Jahan M., Rong H.P., Hong C.N., Pol V.G. (2017). Biomineralization-inspired crystallization of monodisperse α-Mn_2_O_3_ octahedra and assembly of high-capacity lithium-ion battery anodes. J. Mater. Chem. A.

[3] Tang J.L., Lugo C.E.Z., Guzman S.F.A., Daniel G., Kessler V.G., Seisenbaeva G.A., Pol V.G. (2016). Pushing the theoretical capacity limits of iron oxide anodes: Capacity rise of γ-Fe_2_O_3_ nanoparticles in lithium-ion batteries. J. Mater. Chem. A.

[4] Tang J.L., Dysart A.D., Kim D.H., Saraswat R., Shaver G.M., Pol V.G. (2017). Fabrication of Carbon/Silicon Composite as Lithium-ion Anode with Enhanced Cycling Stability. Electrochim. Acta.

[5] Abuzeid H.M., Hashem A.M., Kaus M., Knapp M., Indris S., Ehrenberg H., Mauger A., Julien C.M. (2018). Electrochemical performance of nanosized MnO_2_ synthesized by redox route using biological reducing agents. J. Alloy. Compd..

[6] Cheng F., Chen J., Gou X., Shen P. (2005). High-Power Alkaline Zn–MnO_2_ Batteries Using γ-MnO_2_ Nanowires/Nanotubes and Electrolytic Zinc Powder. Adv. Mater..

[7] Wang S., Liu Q., Yu J., Zeng J. (2012). Anisotropic expansion and high rate discharge performance of V-doped MnO_2_ for Li/MnO_2_ primary battery. Int. J. Electrochim. Sci..

[8] Chen J.B., Wang Y.W., He X.M., Xu S.M., Fang M., Zhao X., Shang Y.M. (2014). Electrochemical properties of MnO_2_ nanorods as anode materials for lithium ion batteries. Electrochim. Acta.

[9] Barbato S., Restovic A., Gautier J.L. (2001). Hollandite cathodes for lithium ion batteries. 1. Synthesis and characterization of the MM′Mn_7_O_16_ compounds (M′ = K., Ba; M′ = Mg, Mn, Fe, Ni). Bol. Soc. Chil. Quim..

[10] Li L., Raji A.R.O., Tour J.M. (2013). Graphene-Wrapped MnO_2_–Graphene Nanoribbons as Anode Materials for High-Performance Lithium Ion Batteries. Adv. Mater..

[11] He Y., Chen W., Li X., Zhang Z., Fu J., Zhao C., Xie E. (2012). Freestanding three-dimensional graphene/MnO_2_ composite networks as ultralight and flexible supercapacitor electrodes. ACS Nano.

[12] Xia H., Lai M., Lu L. (2010). Nanoflaky MnO_2_/carbon nanotube nanocomposites as anode materials for lithium-ion batteries. J. Mater. Chem..

[13] Su Y., Zhang J., Liu K., Huang Z., Ren X., Wang C.A. (2017). Simple synthesis of a double-shell hollow structured MnO_2_@TiO_2_ composite as an anode material for lithium ion batteries. RSC Adv..

[14] Zhang N., Ding Y., Zhang J., Fu B., Zhang X., Zheng X., Fang Y. (2017). Construction of MnO_2_ nanowires@ Ni_1−*x*_Co*_x_*O*_y_* nanoflake core-shell heterostructure for high performance supercapacitor. J. Alloy. Compd..

[15] Liu C., Peng T., Wang C., Lu Y., Yan H., Luo Y. (2017). Three-dimensional ZnFe_2_O_4_@MnO_2_ hierarchical core/shell nanosheet arrays as high-performance battery-type electrode materials. J. Alloy. Compd..

[16] Feng L.L., Xuan Z.W., Zhao H.B., Bai Y., Guo J.M., Su C.W., Chen X.K. (2014). MnO_2_ prepared by hydrothermal method and electrochemical performance as anode for lithium-ion battery. Nanoscale Res. Lett..

[17] Li B.X., Rong G.X., Xie Y., Huang L.F., Feng C.Q. (2006). Low-temperature synthesis of α-MnO_2_ hollow urchins and their application in rechargeable Li^+^ batteries. Inorg. Chem..

[18] Dai J.X., Li S.F.Y., Siow K.S., Gao Z.Q. (2000). Synthesis and characterization of the hollandite-type MnO_2_ as a cathode material in lithium batteries. Electrochim. Acta.

[19] Liu H.D., Hu Z.L., Su Y.Y., Hu R., Tian L.L., Zhang L., Ruan H.B. (2016). Facile Preparation of 1D α-MnO_2_ as Anode Materials for Li-ion Batteries. Int. J. Electrochem. Sci..

[20] Walanda D.K., Lawrance G.A., Donne S.W. (2005). Hydrothermal MnO_2_: Synthesis, structure, morphology and discharge performance. J. Power Sources.

[21] Kijima N., Sakata Y., Takahashi Y., Akimoto J., Kumagai T., Igarashi K., Shimizu T. (2009). Synthesis and lithium ion insertion/extraction properties of hollandite-type MnO_2_ prepared by acid digestion of Mn_2_O_3_. Solid State Ion..

[22] Vernardou D., Kazas A., Apostolopoulou M., Katsarakis N., Koudoumas E. (2016). Hydrothermal Growth of MnO_2_ at 95 °C as an anode material. Int. J. Thin Films Sci. Technol..

[23] Xiao W., Wang D.L., Lou X.W. (2010). Shape-Controlled Synthesis of MnO_2_ Nanostructures with Enhanced Electrocatalytic Activity for Oxygen Reduction. J. Phys. Chem. C.

[24] Ma Z.F., Zhao T.B. (2016). Reduced graphene oxide anchored with MnO_2_ nanorods as anode for high rate and long cycle Lithium ion batteries. Electrochim. Acta.

[25] Luo Y.L. (2007). Preparation of MnO_2_ nanoparticles by directly mixing potassium permanganate and polyelectrolyte aqueous solutions. Mater. Lett..

[26] Raj B.G.S., Asiri A.M., Qusti A.H., Wu J.J., Anandan S. (2014). Sonochemically synthesized MnO_2_ nanoparticles as electrode material for supercapacitors. Ultrason Sonochem..

[27] Zhang X., Yu P., Wang D.L., Ma Y.W. (2010). Controllable Synthesis of alpha-MnO_2_ Nanostructures and Phase Transformation to β-MnO_2_ Microcrystals by Hydrothermal Crystallization. J. Nanosci. Nanotechnol..

[28] Bystrom A., Bystrom A.M. (1950). The Crystal Structure of Hollandite, the Related Manganese Oxide Minerals, and α-MnO_2_. Acta Crystallogr..

[29] Hicks A.L., Dysart A.D., Pol V.G. (2018). Environmental impact, life cycle analysis and battery performance of upcycled carbon anodes. Environ. Sci. Nano.

[30] Liang G., Qin X., Zou J., Luo L., Wang Y., Wu M., Zhu H., Chen G., Kang F., Li B. (2018). Electrosprayed silicon-embedded porous carbon microspheres as lithium-ion battery anodes with exceptional rate capacities. Carbon.

[31] Peng B., Xu Y., Wang X., Shi X., Mulder F.M. (2017). The electrochemical performance of super P carbon black in reversible Li/Na ion uptake. Sci. China Phys. Mech. Astron..

[32] Kim K., Adams R.A., Kim P.J., Arora A., Martinez E., Youngblood J.P., Pol V.G. (2018). Li-ion storage in an amorphous, solid, spheroidal carbon anode produced by dry-autoclaving of coffee oil. Carbon.

[33] Kim K., Lim D.G., Han C.W., Osswald S., Ortalan J.P., Youngblood J.P., Pol V.G. (2017). Tailored Carbon Anodes Derived from Biomass for Sodium-Ion Storage. ACS Sustain. Chem. Eng..

[34] Drewett N.E., Aldous I.M., Zou J.L., Hardwick L.J. (2017). In situ Raman spectroscopic analysis of the lithiation and sodiation of antimony microparticles. Electrochim. Acta.

[35] Yang L., Cheng S., Ji X., Jiang Y., Zhou J., Liu M. (2015). Investigations into the origin of pseudocapacitive behavior of Mn_3_O_4_ electrodes using in operando Raman spectroscopy. J. Mater. Chem. A.

[36] Julien C., Massot M., Baddour-Hadjean R., Franger S., Bach S., Pereira-Ramos J. (2003). Raman spectra of birnessite manganese dioxides. Solid State Ion..

