# Supplementary material for: Basic Medium Heterogeneous Solution Synthesis of α-MnO2 Nanoflakes as an Anode or Cathode in Half Cell Configuration (vs. Lithium) of Li-Ion Batteries
Source: Nanomaterials (Basel). 2018 Aug 9;8(8):608. doi: 10.3390/nano8080608 (PMC6116270; doi:10.3390/nano8080608)
Supplement: Supplementary file 1 [file nanomaterials-08-00608-s001.pdf]

nanomaterials

# Supplementary materials

## *Basic Medium Heterogeneous Solution Synthesis of α-$MnO_2$ Nanoflakes as an Anode or Cathode in half Cell Configuration (vs Lithium) of Li-ion Batteries*

Kyungho Kim,[a] Geoffrey Daniel,[b] Vadim G. Kessler,[c] Gulaim A Seisenbaeva, *[c] Vilas G. Pol*[d]

[a] *Materials Science and Engineering, Purdue University - West Lafayette, West Lafayette, IN, USA*

[b] *Department of Molecular Sciences, Swedish University of Agricultural Sciences, Box 7015, SE-75007 Uppsala, Sweden*

[c] *Department of Forest Products, Swedish University of Agricultural Sciences, Box , SE-75007 Uppsala, Sweden*

[d] *Davidson School of Chemical Engineering, Purdue University, West Lafayette, 47906, IN, USA*

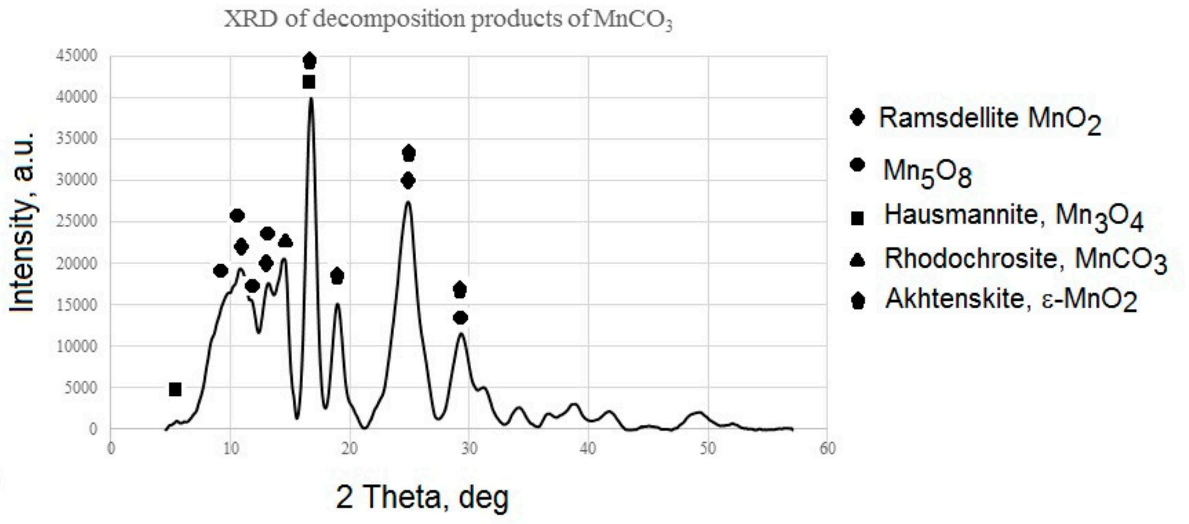


Figure S1. XRD pattern of decomposition products of $MnCO_3$ with characteristic peaks for Ramsdellite $MnO_2$ (ICPDS Card 82-2169), $Mn_5O_8$ (39-1218), Hausmannite $Mn_3O_4$ (80-382), Akhtenskite ε-$MnO_2$ (30-0820) and Rhodochrosite $MnCO_3$ (86-0173) marked in the image.

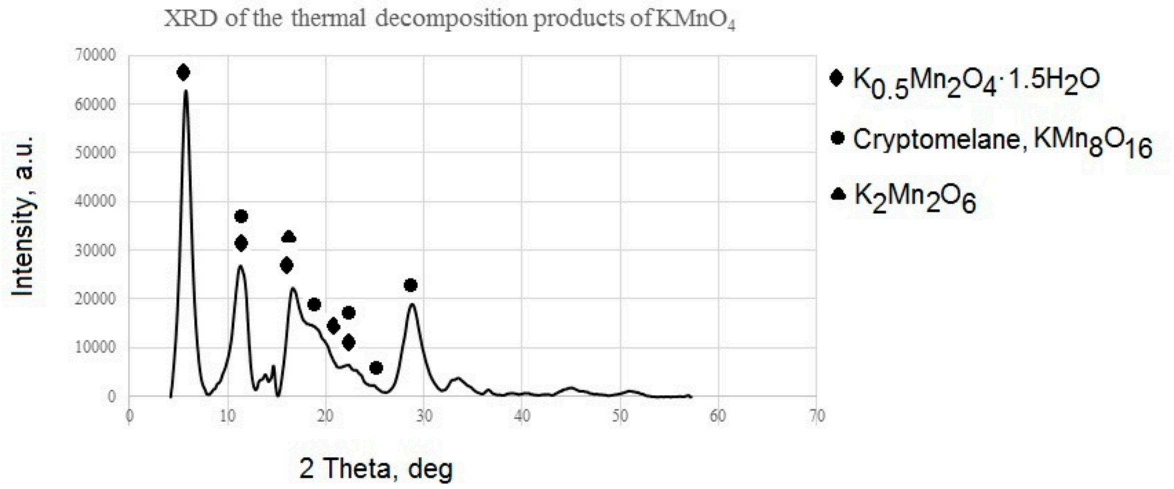


Figure S2. XRD of the material obtained by heat-treatment of the precipitate from the reaction $KMnO_4$ with $NH_3$ in aqueous medium at 600°C in 2h in air with characteristic peaks indicated for $K_{0.5}Mn_2O_4{\cdot}1.5H_2O$ (JCPDS Card 42-1317), Cryptomelane $KMn_8O_{16}$ (34-0168) and $K_6Mn_2O_6$ (ICDD PDF 01-070-1271).

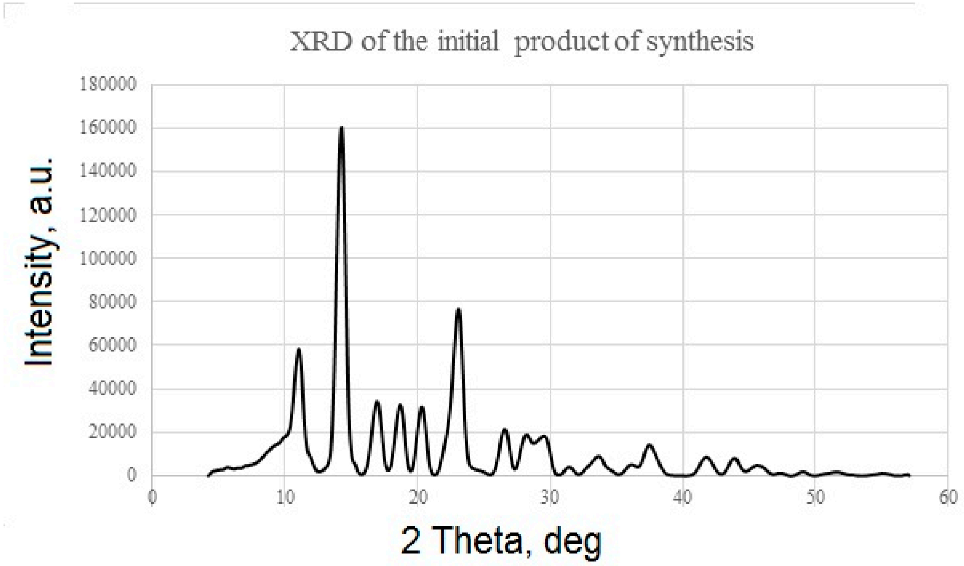


Figure S3. XRD of the initial product of solution synthesis featuring the pure $MnCO_3$ phase ICPDS Card No. 86-0173.

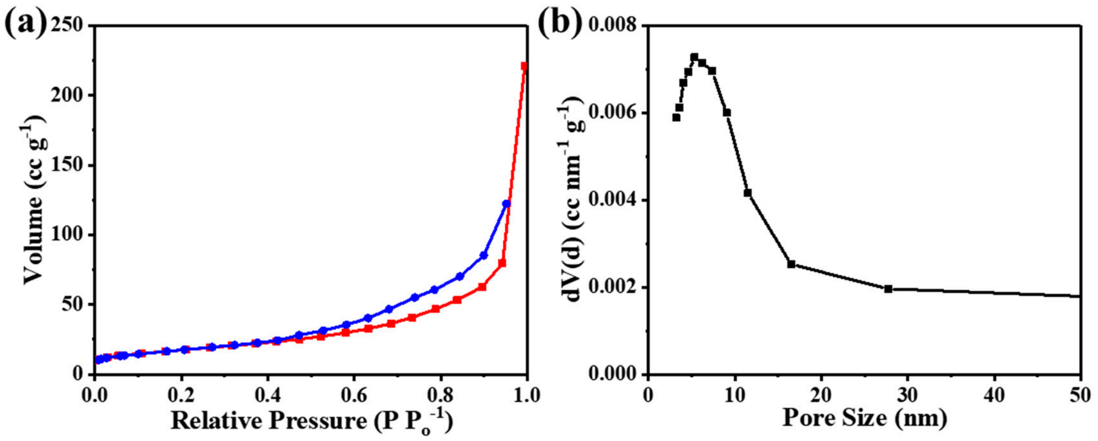


Figure S4. BET analysis of (a) $N_2$ absorption isotherm and (b) pore size distribution profile of prepared α-$MnO_2$ nanomaterial.

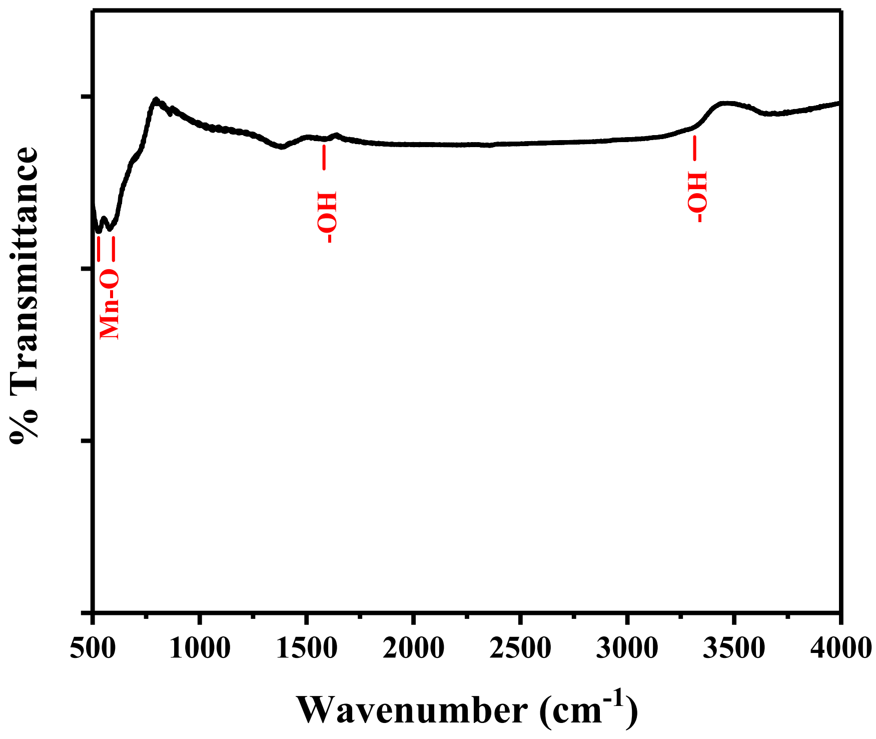


Figure S5. FTIR analysis for produced α-$MnO_2$ nanomaterial.

**A**

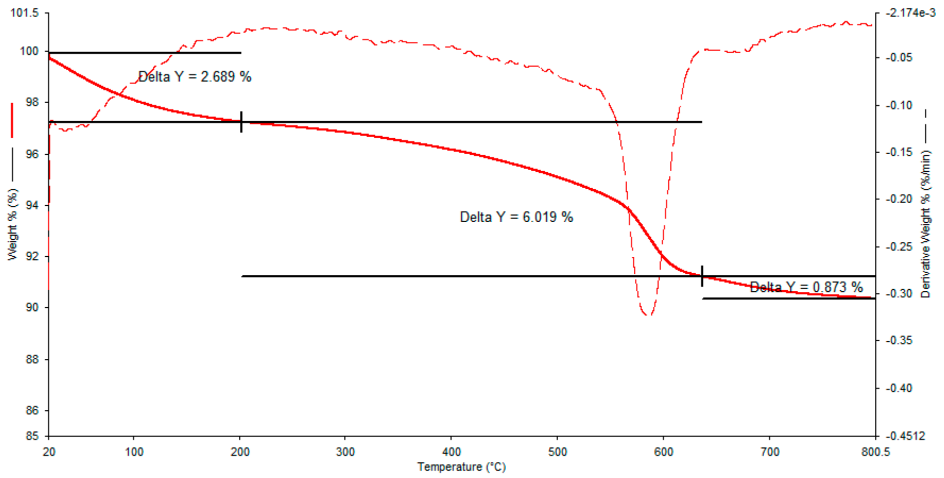


**B**

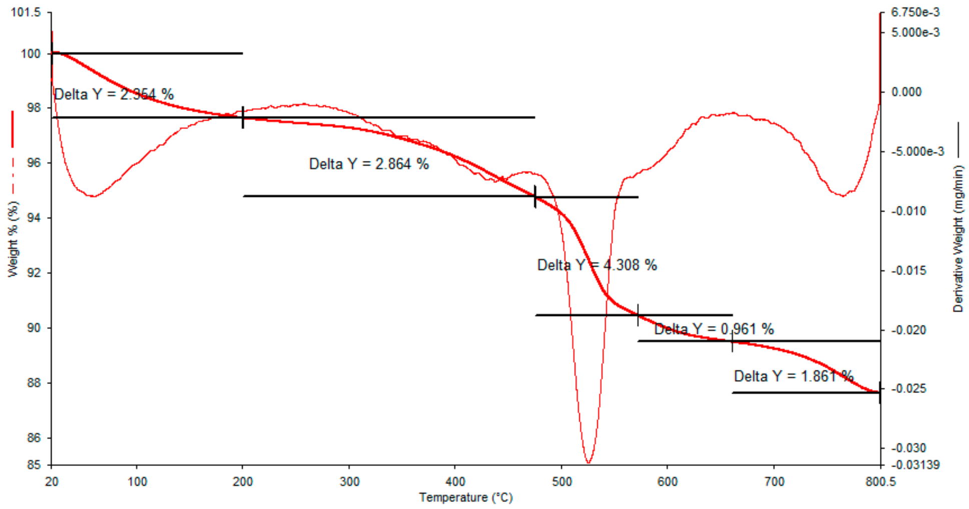


Figure S6. TGA (solid line) and DTG (dashed line) analysis of produced α-$MnO_2$ nanomaterial in the air (A) and in the nitrogen (B) atmosphere.
